# Comparison of Treatment for Metabolic Disorders Associated with Autism:Reanalysis of Three Clinical Trials

**DOI:** 10.3389/fnins.2018.00019

**Published:** 2018-02-12

**Authors:** Leanna M. Delhey, Marie Tippett, Shannon Rose, Sirish C. Bennuri, John C. Slattery, Stepan Melnyk, S. Jill James, Richard E. Frye

**Affiliations:** ^1^Arkansas Children's Research Institute, Little Rock, AR, United States; ^2^Department of Epidemiology, Fay W. Boozman College of Public Health, University of Arkansas for Medical Sciences, Little Rock, AR, United States; ^3^Department of Pediatrics, University of Arkansas for Medical Sciences, Little Rock, AR, United States; ^4^University of Arizona College of Medicine, Phoenix, AZ, United States; ^5^Phoenix Children's Hospital, Phoenix, AZ, United States

**Keywords:** autism, folinic Acid, glutathione, methylation, methylcobalamin, oxidative stress, tetrahydrobiopterin

## Abstract

Autism spectrum disorder (ASD) affects about 1 in 45 individuals in the United States, yet effective treatments are yet to be defined. There is growing evidence that ASD is associated with abnormalities in several metabolic pathways, including the inter-connected folate, methylation and glutathione pathways. Several treatments that can therapeutically target these pathways have been tested in preliminary clinical trials. The combination of methylcobalamin (mB12) with low-dose folinic acid (LDFA) and sapropterin, a synthetic form of tetrahydrobiopterin (BH4) have been studied in open-label trials while high-dose folinic acid has been studied in a double-blind placebo controlled trial. All of these treatments have the potential to positively affect folate, methylation and glutathione pathways. Although the effect of mB12/LDFA and BH4 on methylation and glutathione metabolism have been examined in the open-label studies, these changes have not been compared to controls who received a placebo in order to account for the natural variation in the changes in these pathways. Furthermore, the recent study using high-dose folinic acid (HDFA) did not analyze the change in metabolism resulting from the treatment. Thus, we compared changes in methylation and glutathione metabolism and biomarkers of chronic oxidative stress as a result of these three treatments to individuals receiving placebo. In general, mB12/LDFA treatment had a significant effect on glutathione and cysteine metabolism with a medium effect size while BH4 had a significant effect on methylation and markers of chronic oxidative stress with a large effect size. HDFA treatment did not significantly influence biomarkers of methylation, glutathione or chronic oxidative stress. One caveat was that participants in the mB12/LDFA and BH4 studies had significantly worse markers of glutathione metabolism and chronic oxidative stress at baseline, respectively. Thus, the participants selected in these two clinical trials may have been those with the most severe metabolic abnormalities and most expected to respond to these treatments. Overall this study supports the notion that metabolic abnormalities in individuals with ASD may be amenable to targeted treatments and provide some insight into the mechanism of action of these treatments.

## Introduction

Autism spectrum disorder (ASD) is a behaviorally defined disorder that is estimated to affect up to 1 in 45 individuals in the United States (Zablotsky et al., 2014). Although decades of research has investigated the etiology of this disorder, the etiology remains largely unknown. Epidemiological research suggests that ASD arises from complicated interactions between genetics and environment, but the precise mechanism by which this occurs remains unclear (Hallmayer et al., 2011; Sandin et al., 2014).

Over the last two decades, there has been growing evidence that ASD is associated with impairments in key fundamental physiological processes of the body (Rossignol and Frye, 2012a). Abnormalities which have been verified in multiple studies include impairments in mitochondrial function (Frye and Rossignol, 2011; Rossignol and Frye, 2012a,b), abnormalities in key nutrients and cofactors such as carnitine (Gargus and Lerner, 1997; Filipek et al., 2004; Celestino-Soper et al., 2012; Frye et al., 2013d), folate (Frye et al., 2013e), and tetrahydrobiopterin (Frye, 2010; Frye et al., 2010, 2013b) as well as oxidative stress with associated abnormalities in glutathione-mediated redox regulation (James et al., 2004, 2006, 2009b; Rose et al., 2012a,b; Frye et al., 2013a). What is hopeful about abnormalities in metabolic pathways is that metabolic abnormalities often can be corrected with well-tolerated treatments that either replace key metabolites or provide intermediates that by-pass the metabolic block (Frye and Rossignol, 2014, 2016; Niyazov et al., 2016; Delhey et al., 2017). Such treatments could substantially improve the lives of children with ASD as they target pathophysiological abnormalities, thus correcting underlying abnormalities in faulty biological processes rather than simply treating symptoms.

However, just because metabolic abnormalities are associated with a disorder such as ASD, it does not mean that they are causative or that even correcting them may have an effect. That is, an important caveat is that they could be correlational with ASD and merely an epiphenomenon rather than causational to biological processes associated with ASD symptomatology. One way of providing evidence that these metabolic abnormalities may be integral to important pathophysiological processes is to determine if correcting them will improve ASD symptoms.

**Figure 1:**
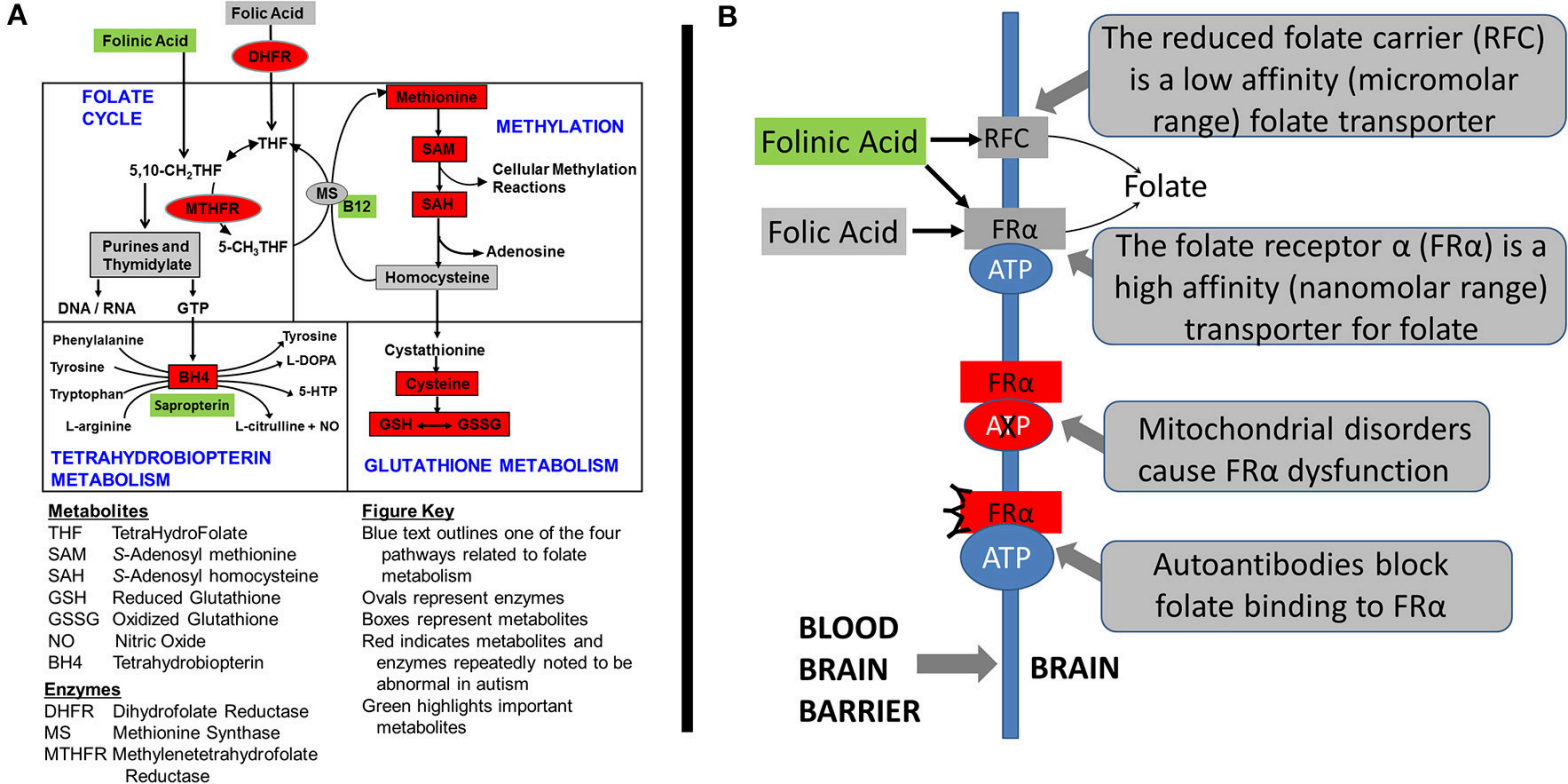
Metabolic pathways disrupted in autism spectrum disorder (ASD). **(A)** The four interconnected critical folate-related metabolic pathways that manifest abnormalities in individuals with autism spectrum disorder: folate, methylation, glutathione and tetrahydrobiopterin pathways. Green boxes represent the treatments examined in this study along with their theoretical targets. Red color indicates metabolites that have been found to be abnormal in children with ASD in multiple studies from multiple laboratories. Boxes represent metabolites and ovals represent enzymes. **(B)** The mechanisms of folate transportation into the brain can be disrupted because of autoantibodies or mitochondrial disorders which result in dysfunction of the folate receptor alpha. Reduced folates, such as folinic acid, can use an alternative transport mechanism known as the reduced folate carrier (RFC).

The authors have investigated three treatments to correct metabolic abnormalities which have demonstrated improvement in behavior and/or metabolism, namely the combination of methylcobalamin (mB12) with low-dose folinic acid (LDFA) to treat oxidative stress in an open-label study (James et al., 2009a; Frye et al., 2013c), sapropterin (BH4) to normalize tetrahydrobiopterin metabolism in an open-label study (Frye et al., 2013b) and high-dose folinic acid (HDFA) to normalize central folate metabolism in a double-blind placebo-controlled study (Frye et al., 2016b). Figure 1A demonstrates where these agents are believed to act within four major interconnected metabolic pathways that are known to be disrupted in ASD. The current study will examine the empirical evidence supporting the metabolic targets of these treatments.

The association of dysfunction in the aforementioned pathways and the effectiveness of these aforementioned treatments have been supported by the studies from others. For example, both mutations (Nashabat et al., 2017) and polymorphisms (James et al., 2006) in the cobalamin receptor has been associated with the development of ASD and individuals with ASD have been shown to have decreased concentrations of cobalamin in the brain (Zhang et al., 2016). Interestingly, the age-dependent decrease in methionine synthase, the target of mB12 in the methionine pathway, may be accelerated in the ASD brain (Muratore et al., 2013). Treatment with cobalamin has been shown to be therapeutic in ASD in both case (Pineles et al., 2010; Corejova et al., 2015) and double-blind placebo-controlled (Bertoglio et al., 2010; Hendren et al., 2016) studies.

As has been summarized previously, early studies in ASD demonstrated central deficiencies in BH4 and both controlled and open-label trials demonstrate a therapeutic effect of sapropterin in children with ASD (Frye et al., 2010). More recent studies have studied patients with ASD and central BH4 deficiency (Frye, 2010) and a recent double-blind placebo-controlled trial has further verified the therapeutic effect of sapropterin (Klaiman et al., 2013). Both sapropterin and cobalamin are believed to target abnormal redox metabolism, oxidative stress and oxidative damage to protein, lipid and DNA that has been repeated documented to be associated with ASD in many studies (Frustaci et al., 2012).

Some of the first cases of cerebral folate deficiency (CFD) were identified to have features of ASD (Ramaekers et al., 2005). Further case-reports and case-series suggested that abnormalities in cerebral folate metabolism was isolated to ASD patients who were low-functioning or had several neurological abnormalities (Moretti et al., 2005, 2008; Ramaekers et al., 2007). Interestingly, these studies documented substantial improvement in ASD and neurological symptoms in these children with ASD when they were treated with HDFA. More recent studies have suggested the number of children with ASD that may manifest disruption in central folate metabolism may not be limited to those who are low-functioning or who have neurological symptoms (Frye et al., 2013e, 2016a) and that a substantial number of children with ASD may benefit from HDFA (Frye et al., 2016b). Figure 1B demonstrates the target of HDFA in the context of CFD.

In all three of these aforementioned clinical treatment studies performed by the authors, biomarkers of redox and methylation metabolism were measured. In the first two studies, changes in redox and methylation metabolism were reported but because the studies were open-label the changes in these biomarkers were not compared to a placebo group. Additionally, in the latter study, the changes in redox and methylation metabolism was not analyzed in the published paper. Since the latter study had a placebo group which had redox and methylation biomarkers measured at the beginning and end of the intervention, we use this placebo group as a control group for all three studies in order to determine if the changes in redox and methylation metabolism were significant as a result of the three interventions.

## Methods

The three clinical trials have been published so we will briefly describe the methods of each. This study was carried out in accordance with the recommendations of the respective Institutional Review Boards (IRBs), described below with written informed consent from parents of subjects and assent where appropriate. Parents of all subjects gave written informed consent and participants themselves gave assent when appropriate in accordance with the Declaration of Helsinki. The protocol was approved by the IRBs named below. Table 1 provides demographics of the participants who underwent each treatment.

**Table 1:** Demographics of the participants in the four treatment groups.

	N^*^	**Age Range**	**Mean Age (SD)**	**% Female**
Methylcobalamin (mB12)/Low-Dose Folinic Acid (LDFA)	39	2y 8m to 7y 8m	5y 0m (1y 5m)	18% (7/39)
Tetrahydrobiopterin (BH4)	8	4y 2m to 6y 9m	5y 0m (1y 2m)	13% (1/8)
High Dose Folinic Acid (HDFA)	15	4y 2m to 13y 4m	8y 2m (3y 2m)	13% (2/15)
Placebo	20	3y 7m to 12y 8m	6y 8m (2y 10m)	20% (4/20)

* *N represents the number of participants that finished the treatment in the clinical trial and had a blood draw for the biomarkers under study. Thus, the number of participants are lower than the number who completed the clinical trials in most cases since blood was not always obtained from every participant at the end of the trial for various reasons, including the participant refusal*.

### Methylcobalamin (mB12)/low-dose folinic acid (LDFA)

This was a prospective 12-week open-label outpatient treatment trial of subcutaneously injected methylcobalamin (mB12; 75 μg/Kg) along with oral low-dose folinic acid (LDFA; 400mcg) twice a day in children diagnosed with ASD who were screened for metabolic markers of poor methylation and redox metabolism (James et al., 2009a). This study was approved by the IRB of the University of Arkansas for Medical Sciences (UAMS; Little Rock AR).

### Tetrahydrobiopterin (BH4)

This was a prospective 16-week open-label outpatient treatment trial of sapropterin (20mg/kg) for 2–6 year old children with confirmed language and/or social delays, ASD and cerebrospinal fluid BH_4_ concentration within the normal range (≤30 nM/L) (Frye et al., 2013b). This study was approved by the IRB of the University of Texas Health Science Center at Houston (Houston, Texas). Redox and methylation biomarkers were measured at baseline, and at 8 and 16 weeks after the start of treatment. To be consistent with the other two trials where biomarkers were measured at 12 weeks, the values for the 8 and 16 week measurements were averaged together.

### High dose folinic acid (HDFA)

This was a double-blind placebo-controlled outpatient treatment trial of high-dose folinic acid (HDFA) (Leucovorin Calcium 2mg/kg/day, maximum 50mg daily) in two divided doses for children with ASD aged 3 to 14 years of age with language impairment (Frye et al., 2016b). This study was approved by the UAMS IRB. Of the 25 participants entering the placebo arm and the 23 participants entering the HDFA arm, 20 and 15 completed two blood draws as the second blood draw was not absolutely required for all participants.

### Biomarkers

All biomarkers were collected as morning fasting samples prior to medication administration. 4ml of blood was collected into an EDTA-Vacutainer tube, chilled on ice and centrifuged at 1500 × g for 10 minutes at 4°C. Plasma was stored at −80°C and underwent HPLC with electrochemical detection within 2 weeks of collection (Melnyk et al., 1999). Selected biomarkers from the redox and methylation pathways were investigated (See Figure 1). For redox metabolism we investigated total (tGSH) and free (fGSH) reduced glutathione, oxidized glutathione (GSSG), the total and free glutathione redox ratios, total cysteine, glutamyl-cysteine and cysteinyl-glycine. For measurements of oxidative damage we measured 3-nitrotyrosine (3NT), a measure of protein oxidation, and 3-chlorotyrosine (3CT), a measure of oxidative damage due to inflammation (myeloperoxidase activity). For methylation we measured methionine, adenosine, homocysteine, S-adenosyl methionine (SAM), S-adenosyl homocysteine (SAH) and the SAM/SAH ratio, a measure of methylation capacity.

### Statistical analysis

Only participants that completed the required blood draws in the clinical trials were included in this study. No imputation for missing data was conducted and individual participants were eliminated from the analysis if laboratory data was missing. Analyses used SAS version 9.3 mixed-effects regression models to estimate the effect and effect size of the treatment (Laird and Ware, 1982). The models included the effect of time and a random intercept. The analysis is a mixed-model regression which is essentially a repeated measures ANOVA with treatment (between group) and time (within group) factors. The models tested the *a priori* hypothesis that the change in the metabolic biomarker was greater for participants who underwent treatment as compared with the placebo group. This effect is examined by testing the interaction between time and treatment in the model using an alpha < 0.05. This effect is F-distributed. For each significant effect we calculate the Cohen's *d*′ in order to demonstrate the effect size. In general a Cohen's *d*′ effect size between 0.2 and 0.4 is small, between 0.5 and 0.7 is medium and 0.8 or above is large. In general an effect size above 0.4 is desired for any intervention.

## Results

### Baseline differences

Vineland adaptive behavior social, daily living and communication subscales were not significantly different across treatment groups or between treatment groups and placebo. The age of the placebo group was not significantly different than the HDFA or BH4 groups but was slightly but significantly higher than mB12/LDFA group [*t*_(58)_ = 3.07, *p* < 0.01]. Gender was not significantly different across groups.

**Figure 2:**
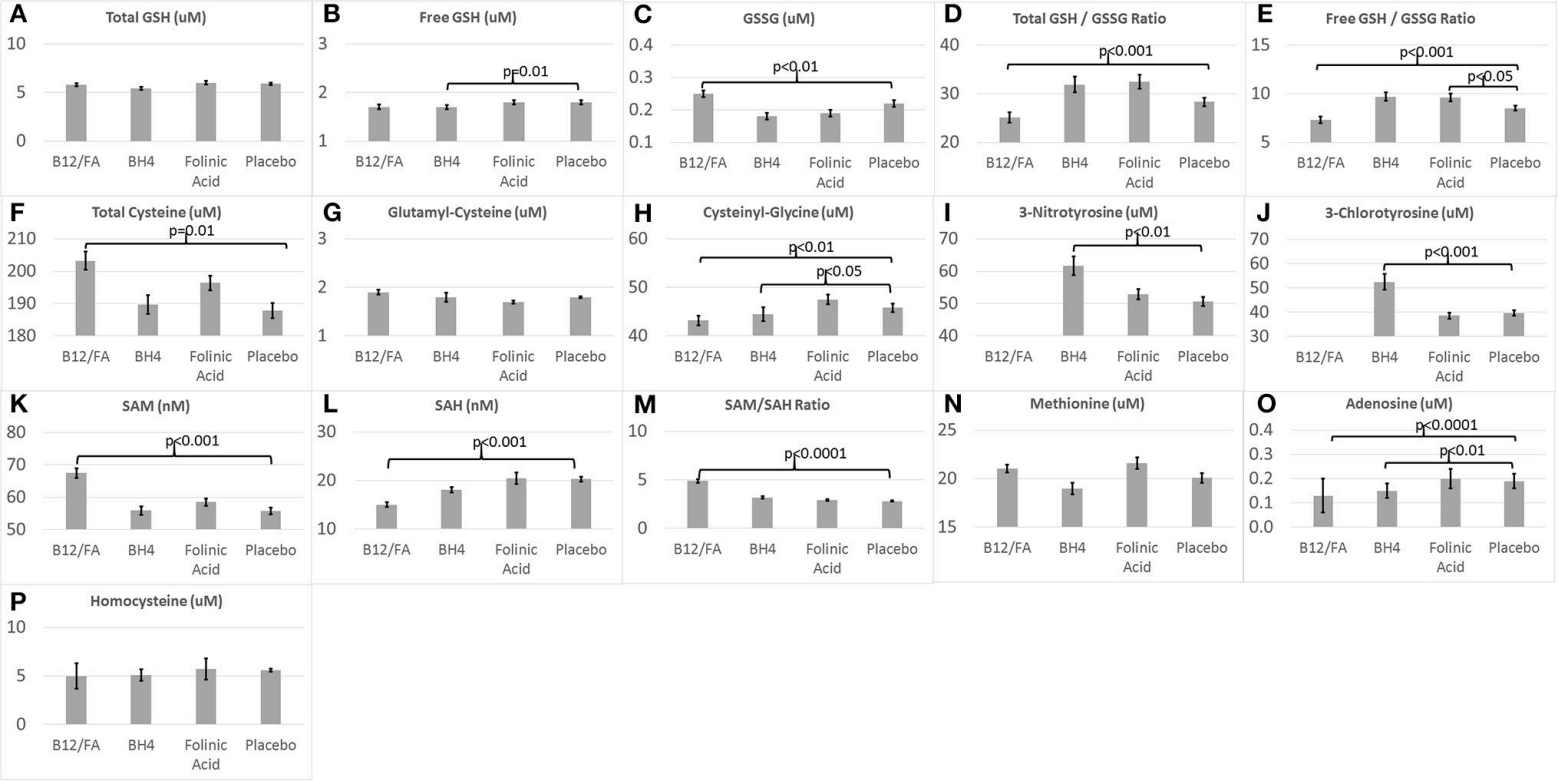
Baseline **(A–H)** glutathione, **(I,J)** chronic oxidative stress, and **(K–P)** methylation biomarkers for the three treatments and placebo.

Figure 2 demonstrates the metabolite baseline values for the three treatment groups and the placebo group.

Baseline values for several biomarkers were significantly different between the mB12/LDFA and placebo groups. GSSG [*F*_(1, 57)_ = 6.53, *p* = 0.01; Figure 2C], total cysteine [*F*_(1, 57)_ = 6.76, *p* = 0.01; Figure 2F], SAM [*F*_(1, 57)_ = 13.67, *p* < 0.001; Figure 2K] and SAM/SAH [*F*_(1, 57)_ = 30.79, *p* < 0.0001; Figure 2M] were significantly higher in the mB12/LDFA group as compared to the placebo group. Total GSH/GSSG [*F*_(1, 55)_ = 15.31, *p* < 0.001; Figure 2D], free GSH/GSSG [*F*_(1, 57)_ = 15.96, *p* < 0.001; Figure 2E], Adenosine [*F*_(1, 57)_ = 15.11, *p* < 0.001; Figure 2O], Cysteinyl-Glycine [*F*_(1, 56)_ = 9.02, *p* < 0.01; Figure 2H] and SAH [*F*_(1, 57)_ = 12.16, *p* < 0.001; Figure 2K] were significantly lower in the mB12/LDFA group as compared to the placebo group at baseline.

**Figure 3:**
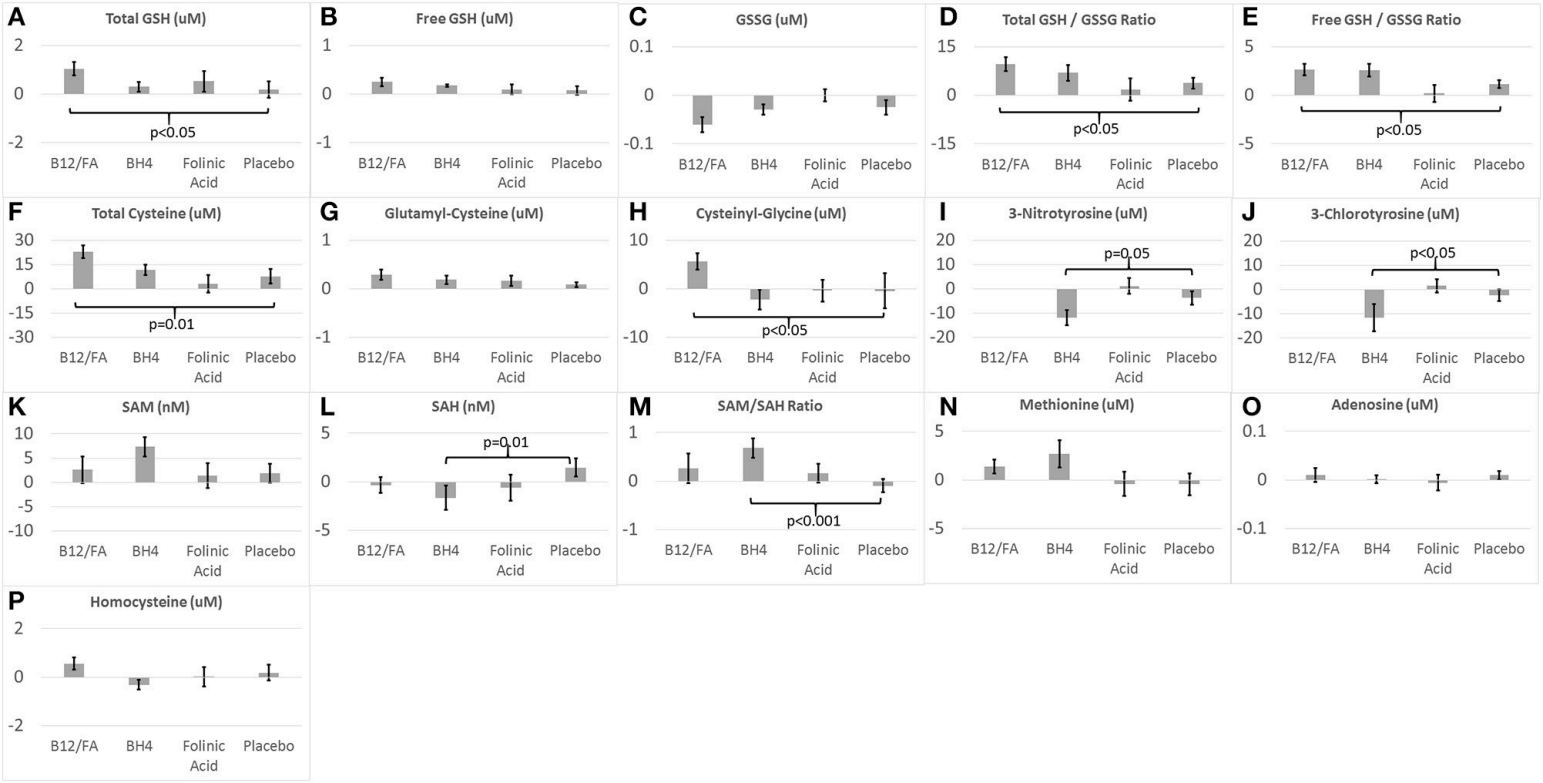
Change in **(A–H)** glutathione, **(I,J)** chronic oxidative stress, and **(K–P)** methylation biomarkers for the three treatments and placebo.

Baseline values for several biomarkers were significantly different between the BH4 and placebo groups. 3NT [*F*_(1, 26)_ = 11.75, *p* < 0.01; Figure 2I] and 3CT [*F*_(1, 26)_ = 17.07, *p* < 0.001; Figure 2J] where higher in the BH4 group as compared to the placebo group while free GSH [*F*_(1, 26)_ = 7.04, *p* = 0.01; Figure 2B], Adenosine [*F*_(1, 26)_ = 8.72, *p* < 0.01; Figure 2O] and Cysteinyl-Glycine [*F*_(1, 26)_ = 5.93, *p* < 0.05; Figure 2H] were lower in the BH4 group as compared to the placebo group.

For the HDFA group, only free GSH/GSSG [*F*_(1, 33)_ = 4.22, *p* < 0.05; Figure 2E] was significantly higher than the placebo group at baseline.

### The effect of treatments on glutathione metabolism

The combination of mB12/LDFA significantly increased total GSH [*F*_(1, 56)_ = 4.23, *p* < 0.05; Cohen's *d*′ = 0.55; Figure 3A], total GSH/GSSG ratio [*F*_(1, 54)_ = 5.76, *p* < 0.05; Cohen's *d*′ = 0.65; Figure 3D] and the free GSH/GSSG ratio [*F*_(1, 56)_ = 4.58, *p* < 0.05; Cohen's *d*′ = 0.57; Figure 3E]. Although the combination of mB12/LDFA decreased GSSG (Figure 3C) and increased free GSH (Figure 3B) these differences did not reach significance. BH4 treatment resulted in changes in glutathione consistent with the combination of mB12/LDFA treatment but only the free GSH/GSSG ratio [*F*_(1, 26)_ = 7.36, *p* = 0.01; Cohen's *d*′ = 1.06; Figure 3E] was statistically significant. HDFA treatment had a negligible effect on glutathione metabolism.

### The effect of treatments on cysteine metabolism

The combination of mB12/LDFA significantly increased total Cysteine [*F*_(1, 57)_ = 6.26, *p* = 0.01; Cohen's *d*′ = 0.66; Figure 3F] and Cysteinyl-Glycine [*F*_(1, 56)_ = 5.45, *p* < 0.05; Cohen's *d*′ = 0.62; Figure 3H] but did not statistically significantly change Glutamyl-Cysteine (Figure 3G) although the change was in the expected direction. BH4 and HDFA did not demonstrate effects on cysteine metabolism.

### The effect of treatments on chronic oxidative stress and inflammation

BH4 treatment significant reduced 3NT [*F*_(1, 26)_ = 4.02, *p* = 0.05; Cohen's *d*′ = 0.78; Figure 3I] and 3CT [*F*_(1, 26)_ = 5.85, Cohen's *d*′ = 0.94; *p* < 0.05; Figure 3J]. HDFA did not appear to have any effect on these biomarkers and these biomarkers were not examined in the mB12/LDFA study.

### The effect of treatments on methylation metabolism

BH4 treatment resulted in a statistically significant decreased in SAH [*F*_(1, 26)_ = 6.95, *p* = 0.01; Cohen's *d*′ = 1.03; Figure 3L] and increase in the SAM/SAH ratio [*F*_(1, 26)_ = 14.17, *p* < 0.001; Cohen's *d*′ = 1.47; Figure 3M]. BH4 treatment did increase SAM but the difference was only borderline statistically significant [*F*_(1, 26)_ = 3.03, *p* = 0.09; Cohen's *d*′ = 0.68; Figure 3K]. mB12/LDFA and HDFA did not significantly change SAM, SAH or the SAM/SAH ratio. BH4 treatment resulted in an increase in methionine but this increase was only borderline significant [*F*_(1, 26)_ = 3.91, *p* = 0.06; Cohen's *d*′ = 0.78; Figure 3N]. Neither mB12/LDFA nor HDFA had a significant effect on methionine. None of the treatments appeared to alter adenosine or homocysteine (Figures 3O,P).

## Discussion

**Figure 4:**
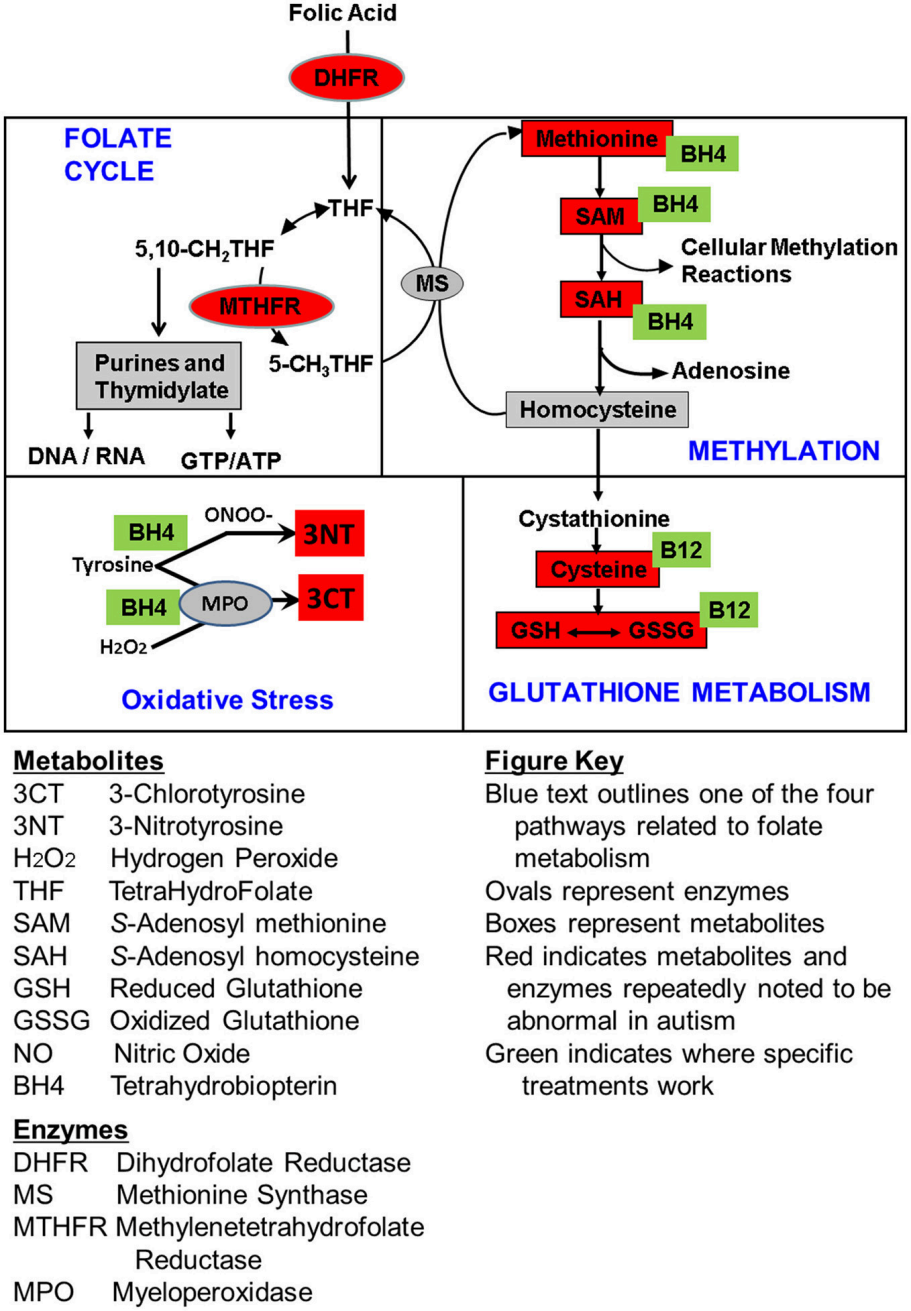
Summary of the empirically determined metabolic targets of treatments evaluated in this study. Green boxes represent the treatments examined in this study. Red color indicates metabolites that have been found to be abnormal in children with ASD in multiple studies from multiple laboratories. Boxes represent metabolites and ovals represent enzymes.

In this study we examined three clinical treatments that have the potential to influence methylation and redox regulation pathways as well as positively affect oxidative stress. Children with ASD were treated with one of these three treatments or placebo and the effect of each treatment over an equivalent 12 week period was compared to the effect of placebo over the same time period. These treatments include mB12/LDFA, BH4 and HDFA. Figure 4 summarizes the metabolites affected by these treatments as well as the pathway for these metabolites. In general mB12/LDFA has a significant influence on redox regulation with medium effect sizes, particularly in the role of positively affecting glutathione metabolism and its precursors, whereas BH4 primarily has a positive effect on markers of oxidative damage as well as methylation metabolism with large effect sizes. Somewhat surprisingly HDFA by itself does not appear to have a major significant effect on redox regulation or methylation pathways possibly because children were not pre-screened for low baseline GSH/GSSG. This provides some empirical evidence into the mechanism of action of these treatments beyond theoretical considerations.

One limitation of examining these clinical trial data in this way is that the ASD population that received the placebo, although similar in development, age and gender, for various reasons, had some differences baseline metabolic characteristics which could affect the effectiveness of the treatment being studied. In some studies, this was by design. For example, the open-label mB12/LDFA study selected individuals with poor glutathione and methylation status to potentiate the ability of the treatment to change these parameters (James et al., 2009a). Interestingly, only glutathione and cysteine metabolism were statistically significantly improved in the mB12/LDFA trial, both with a medium effect size. When compared to the placebo group, which was unselected for metabolic abnormalities, the participants in the mB12/LDFA group demonstrated a more favorable methylation capacity, suggest that they were less severe than what was expected by implementing the pre-selection or that the pathway was up-regulated under conditions of oxidative stress. The mB12/LDFA group did demonstrate less favorable glutathione metabolism than the placebo group at baseline, suggest that the pre-selection process was useful for obtaining children with the worse glutathione metabolism abnormalities. This study confirms that the group of children with ASD and glutathione abnormalities were indeed improved with mB12/LDFA treatment as previously hypothesized and supported by the behavioral data (Frye et al., 2013c). The medium effect size suggests that this is an acceptable therapeutic intervention.

Like the participants in the mB12/LDFA study, participants in the BH4 study demonstrated some less favorable biomarkers at baseline, but these biomarkers were related to chronic oxidative stress and inflammation. These biomarkers related to chronic oxidative stress and inflammation were significantly abnormal at baseline in the BH4 treatment participants as compared to those who received placebo. Thus, the data from this study support the notion that BH4 can improve oxidative damage and methylation abnormalities in children with ASD with a large effect size, with the caveat that this effect may be more pronounced in those with the more severe abnormalities at baseline.

The fact that folinic acid, even at high dose, did not change metabolic markers of methylation and/or glutathione metabolism unless it was combined with mB12 is an important finding as it has direct implication for treatment of inter-related but distinct metabolic disorders associated with ASD. HDFA treatment targets the blockage in the transportation of folate into the brain through the folate receptor alpha caused by blocking or binding autoantibodies (Frye et al., 2013e, 2016a,b, 2017a,b) and is not intended to target methylation or glutathione metabolism directly. These data support the notion that folinic acid needs to be combined with mB12 in order to improve glutathione metabolism.

Overall, this study provides some insight to the potential benefits of safe and well-tolerated therapies that target specific metabolism abnormalities associated with ASD. A better understanding of these physiological abnormalities could lead to the discovery of novel pathways for treatments of potentially many neurodevelopmental disorders and other neurological disorders involving abnormalities in these metabolic pathways.

## Author contributions

LD, MT, and JS Collected that data and samples. SR, SB, and SM processed the samples and performed the assays. RF and SJ designed the studies. RF drafted the manuscript. All authors read and agreed with the manuscript submission.

### Conflict of interest statement

The authors declare that the research was conducted in the absence of any commercial or financial relationships that could be construed as a potential conflict of interest.
